# Elevated expression of LSD1 (Lysine-specific demethylase 1) during tumour progression from pre-invasive to invasive ductal carcinoma of the breast

**DOI:** 10.1186/1472-6890-12-13

**Published:** 2012-08-24

**Authors:** Nuran Serce, Annette Gnatzy, Susanne Steiner, Henning Lorenzen, Jutta Kirfel, Reinhard Buettner

**Affiliations:** 1 Institute of Pathology, University of Bonn, Sigmund-Freud-Str. 25, Bonn 53127, Germany; 2 Institute of Medical Biometrics, Informatics and Epidemiology, University of Bonn, Sigmund-Freud-Str. 25, Bonn 53127, Germany; 3 Institute of Pathology, University of Cologne, Kerpener Str. 62, Cologne, 50924, Germany

**Keywords:** LSD1, DCIS, IDC

## Abstract

**Background:**

Lysine-specific demethylase1 (LSD1) is a nuclear protein which belongs to the aminooxidase-enzymes playing an important role in controlling gene expression. It has also been found highly expressed in several human malignancies including breast carcinoma. Our aim was to detect LSD1 expression also in pre-invasive neoplasias of the breast. In the current study we therefore analysed LSD1 protein expression in ductal carcinoma in situ (DCIS) in comparison to invasive ductal breast cancer (IDC).

**Methods:**

Using immunohistochemistry we systematically analysed LSD1 expression in low grade DCIS (n = 27), intermediate grade DCIS (n = 30), high grade DCIS (n = 31) and in invasive ductal breast cancer (n = 32). SPSS version 18.0 was used for statistical analysis.

**Results:**

LSD1 was differentially expressed in DCIS and invasive ductal breast cancer. Interestingly, LSD1 was significantly overexpressed in high grade DCIS versus low grade DCIS. Differences in LSD1 expression levels were also statistically significant between low/intermediate DCIS and invasive ductal breast carcinoma.

**Conclusions:**

LSD1 is also expressed in pre-invasive neoplasias of the breast. Additionally, there is a gradual increase of LSD1 expression within tumour progression from pre-invasive DCIS to invasive ductal breast carcinoma. Therefore upregulation of LSD1 may be an early tumour promoting event.

## Background

LSD1 (Lysine-specific demethylase 1) is a key enzyme in posttranslational histone modification [1]. Histone modifications including acetylation, methylation, phosphorylation and ubiquitination play an important role in structural changes of chromatin [2,3]. Chromatin modifying activities are dynamically regulated processes and form epigenetic marks on the histone substrate. Thus, enzymes and especially LSD1 are involved in epigenetic gene deregulation and initiate tumourgenesis [3-5]. So far little is known on the in vivo biochemical mechanism by which LSD1 overexpression is linked to neoplastic cell proliferation. Discovered by in vitro data it was shown that LSD1 interfers to G2-M phase cell-cycle and promotes cell proliferation [6]. LSD1 is localised in the nucleus and characterised by a C-terminal amino oxidase domain (AOD) and by an N-terminal domain. The catalytic centre of LSD1 is the Flavin Adenin Dinucleotide-dependent AOD region. This highly specific catalytic domain leads to demethylation of mono- or dimethylated lysine (Lys4) of histone 3 (H3) [5,7].

LSD1 has been analysed in several human tumour entities. It has been shown to be overproduced in prostate cancer [8,9], neuroblastoma [10], lung, colorectal and bladder cancer [11,12]. Furthermore, LSD1 expression has also been investigated in invasive breast carcinoma. Lim et al. [13] showed a significant positive correlation between overexpression of LSD1 and negative oestrogen receptor status in breast carcinoma. Also there was an inverse correlation between high LSD1 expression levels and low progesterone receptor status. Other histopathological data like tumour size (pT1-pT4), nodal status and human epidermal growth factor receptor *(HER 2)* status reached no significant correlation with LSD1 expression. However in consideration of the fact that breast carcinoma with positive steroid hormone receptor status responds to endocrine treatment [14] and therefore reveals a better prognosis, Lim et al. supposed that upregulation of LSD1 and its correlation with negative oestrogen receptor status could be a biomarker for aggressive tumour biology in breast cancer.

Consistent with its tumour promoting role, the specificity of posttranslational modification conferred by LSD1 has been investigated by Bradley et al. [15]. They analysed human mammary epithelial cell lines after carcinogen exposure and examined equal levels of histone H3 total protein and histone H4, but significantly decreased levels of mono-methyl histone H3 (Lys4) compared with control groups which were not treated. They pointed out that downregulation of histone H3 (Lys4) was related to LSD1 upregulation after carcinogen treatment. In addition accumulated levels of carcinogen exposure were related to accumulated levels of LSD1 expression compared to control human mammary epithelial cell lines. Thus increased LSD1 expression was assumed to be an early step in breast cancer development.

Surprisingly, LSD1 has not been studied in pre-invasive breast cancer lesions, so far. To the best of our knowledge, this is the first study to analyse LSD1 protein expression in pre-invasive breast neoplasias. We systematically examined LSD1 expression in low, intermediate and high grade ductal carcinoma in situ in comparison to invasive ductal carcinoma.

## Methods

### Tissue specimens

Paraffin embedded breast cancer specimens were selected from the tumour data bank of the Institute of Pathology, University of Bonn. Patients` age ranged from 27 to 88 years with a median of 56 years. An experienced surgical pathologist evaluated haematoxylin-eosine stained slides of all specimens. Histologically all DCIS lesions were graded according to established criteria by the World Health Organisation (WHO) [16]. These are based on nuclear grading, necrosis, microcalcification and architecture. DCIS was classified in low grade (n = 27), intermediate grade (n = 30) and high grade (n = 31) and was compared with invasive ductal breast carcinoma specimens (n = 32) (Table 1). 

**Table 1:** Clinicopathological and immunohistochemical parameters in relation to LSD1 immunoreactivity in invasive ductal breast carcinoma

**Variable**	**Categorisation**	**LSD1 immunoreactivity**			
		**n analysable**	**low **^**b**^	**abundant **^**b**^	**p **^**c**^
***Clinicopathological data:***					
Tumour stage^a^					
Tumour stage^a^		22	5	17	0.370
pT2		5	2	3	
pT3		1	0	1	
pT4		1	1	0	
unknown		3			
Lymph node status^a^					
pN0		16	1	15	**0.010**
pN1-3		9	5	4	
unknown		7			
Histological grade					
G1		2	0	2	**0.010**
G2		16	1	15	
G3		14	7	7	
Histological type					
ductal		32			
***Immunohistochemistry (IHC):***					
Oestrogen receptor status					
negative (IRS 0–2)		10	6	4	**0.004**
positive (IRS 3–12)		22	2	20	
Progesterone receptor status					
negative (IRS 0–2)		14	6	8	0.090
positive (IRS 3–12)		18	2	16	
HER2 status					
weak (0-2+)		26	7	19	1.0
strong (3+)		6	1	5	

^a^According to UICC: TNM Classification of Malignant Tumours (7th edition). Sobin LH, Gospodarowicz MK, Wittekind CH (eds) Wiley-Blackwell, Oxford 2009.

^b^LSD1 immunoreactivity: low = IRS 0–9, abundant = IRS 10–12.

^c^Fisher’s exact test (two-sided), bold face representing significant data (*P* <0.05).

All 120 patients gave informed consent for further analysis of their tissue for research purposes and the Instructional Review Board of the participating centre approved the study.

### LSD1 immunohistochemistry

LSD1 immunohistochemistry was done by using an anti-LSD1 antibody (catalog No. 100–1762, Novus Biologicals, Littleton, CO, diluted 1:250). The slides were scored independently by two experienced pathologists (NS and RB) according to the semi-quantiative scoring system suggested by Remmele and Stegner [17] considering staining intensity and percentage of positive cell nuclei. The staining intensity was described by scores between 0 and 3 (0 = no reaction, 1 = low, 2 = moderate, 3 = strong). Accordingly, the number of positive cell nuclei was counted and scored between 0 and 4 (0 = no positive cell nuclei, 1 = <10% positive cell nuclei, 2 = 10-50% positive cell nuclei, 3 = 51-80% positive cell nuclei, 4 = > 80% positive cell nuclei). The product of staining intensity and percentage of positive cell nuclei resulted in a score (IRS) between 0 and 12. Each sample was categorised by this rating score (Table 2). 

**Table 2:** LSD1 expression (median IRS) in DCIS and IDC

**Tissue**	**n**	**LSD1 expression (median IRS)**
low grade DCIS	27	8
intermediate grade DCIS	30	10
high grade DCIS	31	12
IDC	32	12

### Statistical analysis

SPSS software version 18.0 (SPSS GmbH software, Zurich, Switzerland) was used for statistical analysis. Differences were considered statistically significant when p-values were <0.05. A non-parametrically Kruskal-Wallis-H-Test was employed to analyse differences in expression levels. Bonferroni-Holm-procedure was used for analysing single groups in comparison to each other.

## Results

### LSD1 is expressed in pre-invasive and invasive ductal breast carcinoma

LSD1 expression has not been analysed in pre-invasive breast lesions, so far. Therefore we investigated LSD1 in pre-invasive ductal carcinoma in situ in comparison to invasive breast cancer.

**Figure 1:**
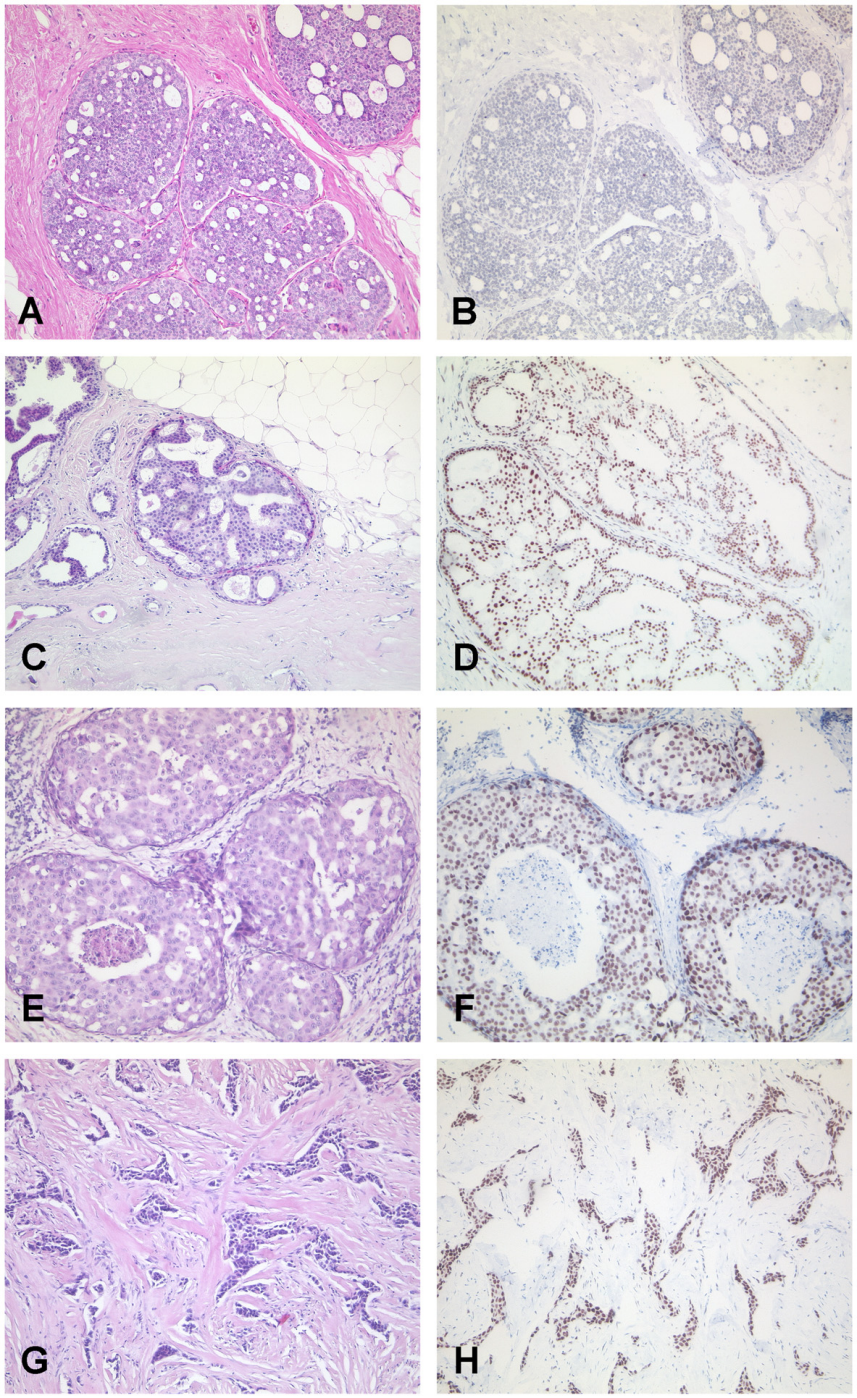
**Immunohistochemical expression of LSD1 in pre-invasive and invasive ductal breast carcinoma. **(**A**, **B**) Low grade DCIS shows weak nuclear LSD1 expression (IRS = 4). (**C**, **D**) In intermediate grade ductal carcinoma in situ LSD1 expression is more intense (IRS = 8) than in low grade ductal breast cancer. (**E**, **F**) In high grade ductal carcinoma in situ LSD1 expression is more abundant (IRS = 12) compared with low grade and intermediate grade ductal carcinoma in situ. (**G**, **H**) In invasive ductal breast carcinoma LSD1 expression is as strong as in high grade ductal carcinoma (IRS = 12). Magnification A-H: 100x.

Using immunohistochemistry we analysed LSD1 expression in low (Figure 1A), intermediate (Figure 1C) and high grade ductal carcinoma in situ (Figure 1E) as well as in invasive ductal carcinoma of the breast (Figure 1G). Immunohistochemical staining revealed nuclear LSD1 expression both in pre-invasive and invasive breast cancer epithelial cells. LSD1 was differently expressed within the pre-invasive lesions and also in comparison to the pre-invasive forms with invasive ductal breast carcinoma.

In low grade DCIS (Figure1B) nuclear LSD1 expression was heterogeneous and weak (IRS = 4). In intermediate grade DCIS staining of LSD1 was more intense (IRS = 8) compared to low grade DCIS (Figure1D). LSD1 was abundantly expressed in high grade DCIS (IRS = 12) (Figure1F) compared to intermediate and low grade DCIS. In invasive ductal breast carcinomas nuclear LSD1 staining was as strong as in high grade ductal carcinoma (IRS = 12) (Figure1H).

### Upregulation of LSD1 expression during tumourgenesis from pre-invasive to invasive ductal breast carcinoma

**Figure 2:**
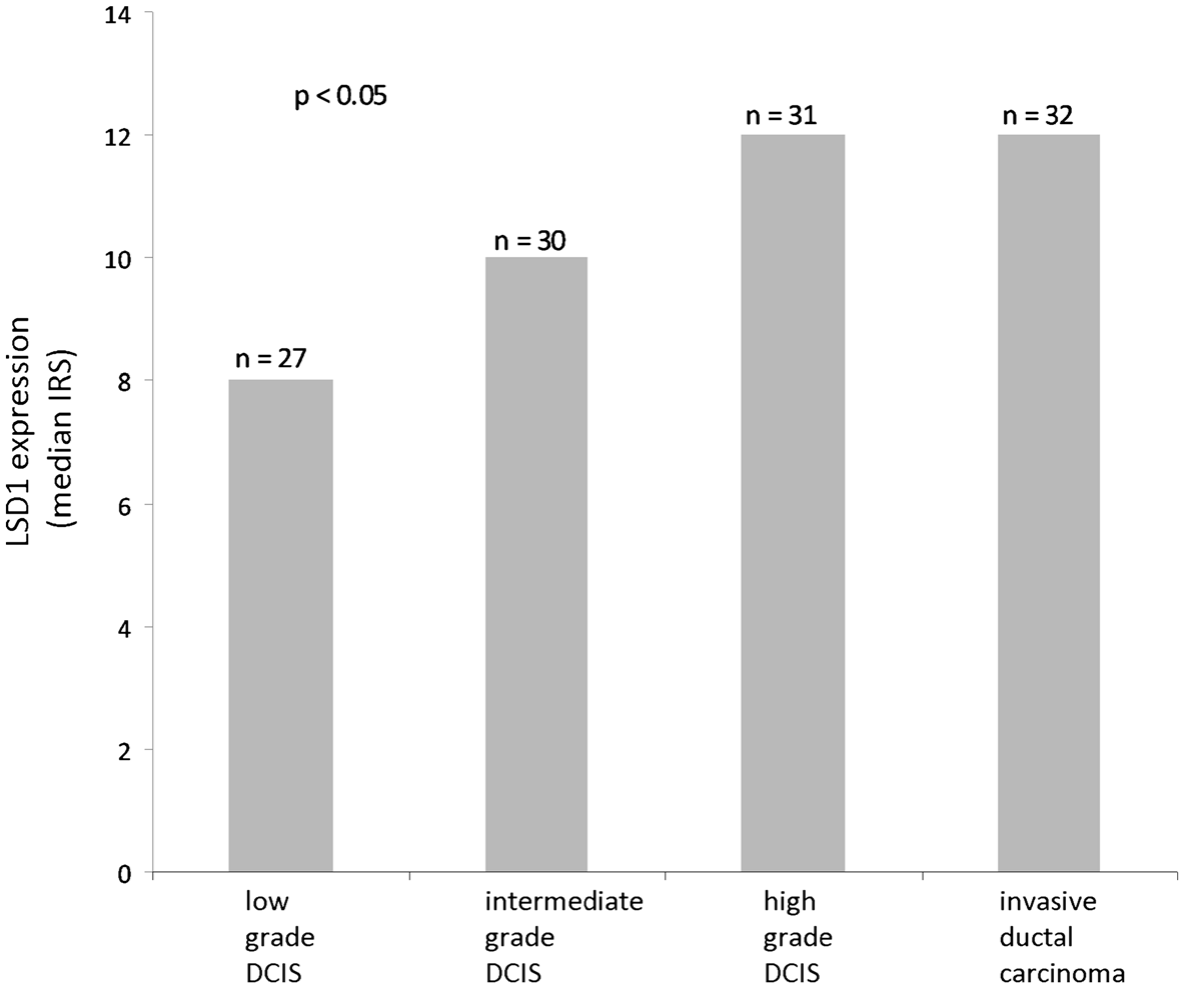
**Elevated expression of LSD1 during tumour progression. **A significant difference in expression during tumour progression from ductal carcinoma in situ (low, intermediate and high grade) to invasive ductal breast cancer was detected (p <0.05, Kruskall-Wallis H-test).

In statistical analysis the median of LSD1 expression level (IRS) was significantly increased during tumour progression from pre-invasive to invasive ductal breast carcinoma (low grade DCIS = 8, intermediate grade DCIS = 10, high grade DCIS = 12, invasive ductal breast carcinoma = 12) (p <0.05, Kruskall-Wallis-H-test) (Table 2, Figure2).

Analysing single groups in comparison to each other we found that LSD1 was significantly overexpressed in high grade DCIS versus low grade DCIS (p <0.001). Differences were also statistically significant between low/intermediate DCIS and high grade DCIS (p <0.032), but also between low/intermediate DCIS and invasive ductal breast carcinoma (p <0.001). Consistently, statistical significance of LSD1 expression was also reached between low grade DCIS and invasive ductal carcinoma (p <0.0001) and low grade DCIS versus high grade DCIS/invasive ductal carcinoma (p <0.0001) (Bonferroni-Holm-procedure).

### Statistical correlation of LSD1 expression in invasive ductal breast carcinoma

LSD1 expression was correlated with clinicopathological and immunohistochemical parameters in invasive ductal breast carcinoma. LSD1 expression was significantly associated with lymph node status, histological grade and with the oestrogen receptor status. There was no siginificant correlation with tumour stage, progesterone receptor status and Her2 expression (Table 1).

## Discussion

LSD 1 is a member of monoaminoxidase enzymes with an important role in controlling gene expression by histone modification [5]. In accordance with the perceived role of LSD1 in cell proliferation, significant expression of LSD1 has been reported in diverse human tumour entities, including breast cancer. Until now, LSD1 expression has not been analysed in pre-invasive breast cancer lesions, so far. In this study we developed a first systematic expression analysis of LSD1 in pre-invasive (n = 88) and invasive (n = 32) breast carcinoma.

Using immunohistochemistry we here show that LSD1 is also expressed in pre-invasive ductal neoplasias of the breast. Additionally, we detected a significant gradual increase of LSD1 expression during tumour progression from low, intermediate and high grade ductal carcinoma in situ to invasive ductal breast carcinoma (p <0.05). Our results implicate that LSD1 could play a key role in breast cancer development. So upregulation of LSD1 may be an early tumour promoting event in breast carcinoma. This is supported by the work of Bradley et al. [15] showing the dynamic movement of LSD1 from the nuclear periphery into the nucleus after carcinogen treatment in human mammary epithelial cell lines. Furthermore increased LSD1 expression levels were found in increasing carcinogen treated human mammary epithelial cell lines compared to non-treated controls. They concluded that upregulation of LSD1 and localisation into the nucleus are mechanisms that are responsible for demethylation of histone H3 (Lys4) by LSD1 affecting genes like p57^kip2^, a cyclin-dependent kinase inhibitor, which is known to be essential in tumourgenesis of breast cancer cells. It is supposed that LSD1 forms complexes with different co-factors and depending on the promoter context it has a gene activating or repression function [1]. A recent study by Scoumanne et al. [6] analysed the role of LSD1 in the human malignant breast cancer cell line MCF7. They found out that LSD1 downregulation decreased the number of proliferating breast cancer cells. Even though the concrete mechanism in which LSD1 is linked to cancer development has not been fully examined, it has been shown that high LSD1 expression is a characteristic feature of cancer cells.

Consistent with these data, we detected high expression levels of LSD1 in invasive ductal breast carcinoma which supports the results of a study by Lim et al. [13] showing abundant LSD1 expression in invasive breast cancer, as well. In conclusion our data imply a positive association between LSD1 overexpression and progression, proliferation as well as increasing invasiveness of breast cancer cells.

However, in our collective of invasive ductal breast carcinoma the inverse correlation of LSD1 expression with lymph node status, histological grade and oestrogen receptor status may be due to a inhomogeneous and relatively small group size of invasive breast carcinoma specimens because this study was not constructed to validate LSD1 expression in invasive breast carcinoma as it was already analysed in a previous work by Lim et al. [13]. Furthermore, our collective of invasive ductal breast carcinomas consists mainly of tumours with small tumour size accordingly pT1 tumours whereas Lim et al. [13] analysed more tumours of a higher stage respectively pT2 to pT4 invasive breast carcinomas. Nevertheless, the association between LSD1 expression and oestrogen receptor status has to be further investigated and validated in greater cohorts of invasive breast carcinomas.

In addition LSD1 could be an interesting target molecule in the treatment of breast carcinoma. Usually, DCIS treatment involves breast conserving surgery by local tumour excision or mastectomy depending on free margins to reduce the risk of ipsilateral recidive. Supplemental radiation is part of adjuvant therapeutic regime [18]. Until now, there has been an absence of common guidelines for the use of hormone therapy or Trastuzumab, a monoclonal antibody, for patients with (human epidermal growth factor) *HER 2* positive DCIS [19-22]. Boughey et al. [19] suggested that anti-hormone therapy should be part of adjuvant therapy regime in oestrogen receptor positive DCIS and the use of Trastuzumab for DCIS was seen as an option which needs to be more specified in future. Nonetheless, it was shown by clinical evidence that only patients with positive oestrogen receptor status responded to anti-hormone treatment and a subgroup of patients developed resistance after extended treatment.

Therefore there is a need for alternative drug treatment in case of resistance to anti-oestrogens. With regard to offering other suitable options of breast cancer therapy in connection with other eligible targets, LSD1 may be such a target mark as LSD1 inhibitors were discussed as novel breast cancer therapeutics [13].

Because of its structural analogy with monoaminoxidases, LSD1 was shown to be inhibited by nonspecific monoaminoxidase inhibitors like tranylcypromine [23]. In a previous work of Lee et al. [23] tranylcypromine was found to inhibit embryonal carcinoma cells. Therefore tranylcypromine was discussed as a possible novel anti-cancer target referring to breast cancer, as well. In concordance with this, Lim et al. [13] showed inhibition of LSD1 in breast cancer cells in vitro by tranylcypromine and clorgyline, a selecitve monoaminoxidase inhibitor, leading to inhibitory effect on LSD1 and reduced growth of the breast cancer cell lines MCF7, T47D, MDA-MB 453 and MDA-MB 231.

## Conclusions

In the future LSD1 detection may be an early identification marker of breast cancer and a potential target for early therapeutic strategies. However, in our collective of invasive ductal breast carcinoma the inverse correlation of LSD1 expression with oestrogen receptor status may be due to a inhomogeneous and relatively small group size of invasive breast carcinoma specimens mainly consisting of pT1 tumours [24]. Further studies are underway to analyse the expression and function of LSD1 in a larger cohort of breast cancer specimens including patients` survival data to evaluate the prognostic relevance of LSD1 in breast carcinoma.

## Competing interests

The authors declare that they have no competing interest.

## Authors’ contributions

NS: participated in design and coordination of the study, data analysis, data interpretation, establishment and evaluation of the immunohistochemistry and drafted the manuscript. AG: participated in data analysis, data interpretation, establishment and evaluation of the immunohistochemistry and drafted the manuscript. SS: supported in establishment and evaluation of the immunohistochemistry as well as in data interpretation. HL supported with expertise in data analysis, data interpretation and critically revised the manuscript. JK participated in study coordination, data interpretation and critically revised the manuscript. RB participated in study design, data analysis, data interpretation and drafting of the manuscript. All authors read and approved the final manuscript.

## Funding

The study was supported by a grant (BONFOR program) of the Faculty of Medicine, University of Bonn, to Dr. Nuran Serce.

The study was approved by the ethics committee of the Faculty of Medicine, University of Bonn.

## Pre-publication history

The pre-publication history for this paper can be accessed here:

http://www.biomedcentral.com/1472-6890/12/13/prepub
